# Supplementary material for: The rough sound of salience enhances aversion through neural synchronisation
Source: Nat Commun. 2019 Aug 14;10:3671. doi: 10.1038/s41467-019-11626-7 (PMC6694125; doi:10.1038/s41467-019-11626-7)
Supplement: Supplementary file 1 — Supplementary Information [file 41467_2019_11626_MOESM1_ESM.pdf]

Supplementary Information

The rough sound of salience enhances aversion through neural synchronisation

Arnal et al.

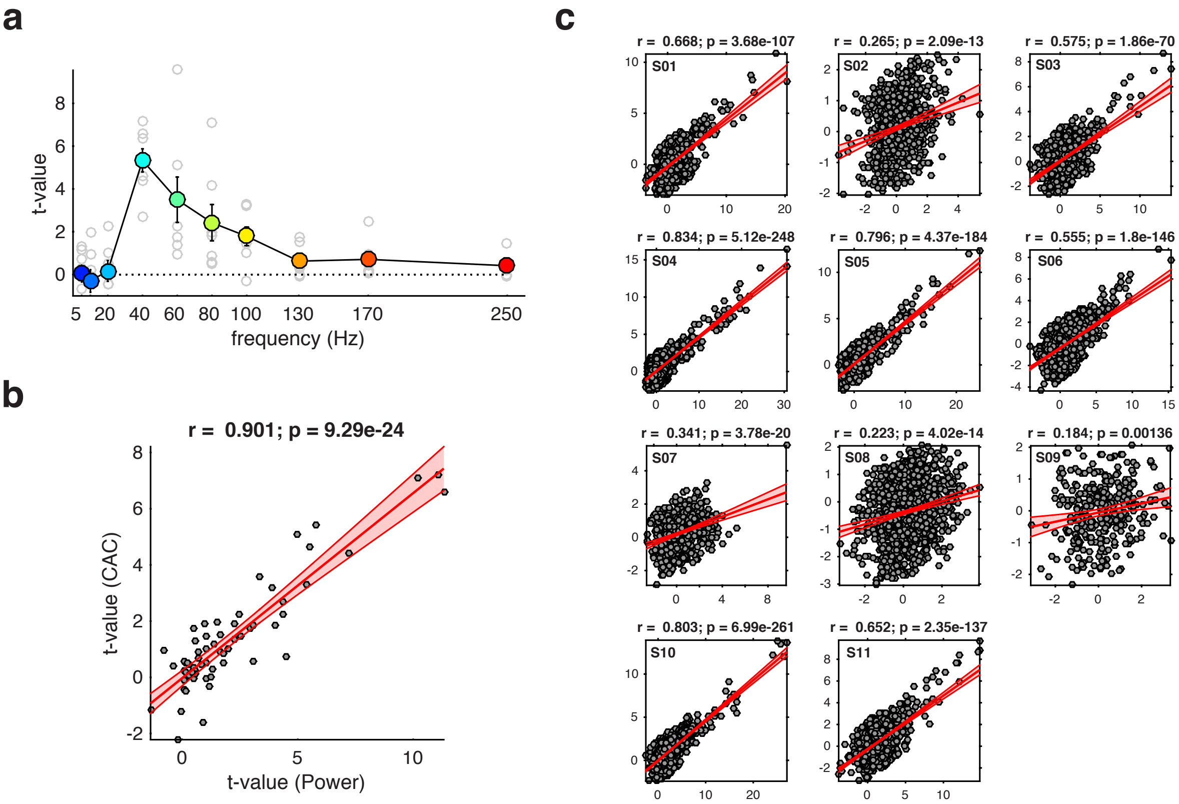

Supplementary Figure 1: Frequency-tagging approaches approximate CAC measurements.

a. Estimation of the power of neural responses in the late peristimulus time window [0.8–1.8s] as a function of stimulus rate using a frequency-tagging approach (instead of CAC as shown in Fig. 2C, bottom graph). Each coloured data point reflects neural response power (averaged across ‘entrained’ electrodes and participants, see Methods section) at the stimulus frequency. Grey circles correspond to individual data.

b. In order to compare the results obtained using CAC and frequency-tagging approaches, we measured the correlation between the outcomes of each measurement type across participants and stimulation frequencies. The strong association between the two measurements (Pearson’s r^2 = 0.811, *i.e.* more than 80% shared variance) suggests that the two approaches consistently capture similar neural steady-state effects.

c. A similar correlative approach was conducted at the individual level across all electrodes and frequencies to compare the outcomes of CAC and frequency-tagging approaches. This analysis reveals that the two methods provide highly similar results. Qualitatively, these observations further suggest that correlation strength between these two approaches depends on individual signal-to-noise ratios (*i.e.* is highest in participants with high t-values ranges).
