# Supplementary material for: The rough sound of salience enhances aversion through neural synchronisation
Source: Nat Commun. 2019 Aug 14;10:3671. doi: 10.1038/s41467-019-11626-7 (PMC6694125; doi:10.1038/s41467-019-11626-7)
Supplement: Supplementary file 2 — Description of Additional Supplementary Files [file 41467_2019_11626_MOESM2_ESM.docx]

Description of Additional Supplementary Files

**Supplementary Movie 1:** CAC spatial response patterns differ across stimulation frequencies. Top panels: Electrodes showing sustained, significant stimulus-brain coherence (CAC) are spatially located in widespread cerebral areas. Colours correspond to the stimulation frequency. Lower plot: CAC as a function of stimulus rate, averaged across significantly ‘entrained’ electrodes and participants. Light grey circles correspond to individual data. Error bars indicate SEM.
